# Functional Evaluation of Awareness in Vegetative and Minimally Conscious State

**DOI:** 10.2174/1874440001711010017

**Published:** 2017-04-27

**Authors:** Silvia Marino, Lilla Bonanno, Rosella Ciurleo, Annalisa Baglieri, Rosa Morabito, Silvia Guerrera, Carmela Rifici, Antonio Giorgio, Placido Bramanti, Nicola De Stefano

**Affiliations:** 1 IRCCS Centro Neurolesi “Bonino Pulejo”, Messina, Italy; 2 Department of Biomedical and Dental Sciences and Morphofunctional Imaging, University of Messina, Italy; 3 UOSA Experimental Neurology, Dept. of Medicine, Surgery & Neuroscience, University of Siena, Italy

**Keywords:** Differential diagnosis, Functional magnetic resonance imaging, Minimally conscious state, Outcome, Vegetative state

## Abstract

**Objective:**

The aim of this study was to assess differences in brain activation in a large sample of Vegetative State (VS) and Minimally Conscious State (MCS) patients, using functional magnetic resonance imaging (fMRI).

**Methods:**

We studied 50 patients four to seven months after brain injury. By using international clinical criteria and validated behavioural scales such as the Glasgow Coma Scale and the Clinical Unawareness Assessment Scale, the patients were grouped into *VS* (n=23) and MCS (n=27). All patients underwent to fMRI examination. After 6 months, the patients were reassessed using Glasgow Outcome Scale and Revised Coma Recovery Scale.

**Results:**

fMRI showed significant (p<0.01, cluster-corrected) brain activation in the primary auditory cortex bilaterally during the acoustic stimuli in patients with both VS and MCS. However, ten patients clinically classified as VS, showed a pattern of brain activation very similar to that of MCS patients. Six months later, these ten VS patients had significant clinical improvement, evolving into MCS, whereas the other VS patients and patients with MCS remained clinically stable.

**Conclusion:**

Brain activity could help in discerning whether the status of wakefulness in *VS* is also accompanied by partial awareness, as occurs in MCS. This may have very important prognostic implications.

## INTRODUCTION

Functional neuroimaging is increasingly used in the clinical domain of Vegetative State (VS) and Minimally Conscious State (MCS) patients [1-4]. Recent applications include protocols designed to monitor a) the natural history of recovery from acquired brain injury, b) to assess the effects of neuro-rehabilitative interventions and c) to better understand these clinical conditions to be able to perform a correct differential diagnosis, as recent neurophysiological studies showed [5, 6]. In fact, in the clinical practice, the assessment of awareness is necessary and the misdiagnosis rate is significantly high.

Using simple noxious somatosensory and auditory stimulation, positron emission tomography (PET) studies have showed in VS patients preserved activation in lower-level primary sensory cortices but not in higher-order associative cortices, secondary somatosensory, insular, posterior parietal, and anterior cingulate cortices, which were otherwise activated in healthy subjects [4, 7-9]. The observation of a functional disconnection from primary auditory areas to limbic areas after auditory stimuli [10] suggests that residual cortical processing in VS patients does not lead to integrative processes, which are thought to be necessary for awareness.

However, these findings have not been confirmed by fMRI studies on MCS and *VS* patients converted to MCS [11-14], where activation of higher-order associative cortices was indeed found. In fact, in a study performed on a group of 14 aetiologically heterogeneous VS and MCS patients using a hierarchical fMRI auditory processing paradigm [13], the two severely disabled but conscious patients showed preserved speech processing at all the levels whereas, contrary to the diagnostic criteria for the VS, three patients demonstrated some evidence of preserved speech processing. In the same study, six months after fMRI examination, all the patients recovered behaviourally with respect to those who did not show comparable brain activations. In the first fMRI study performed on patients with MCS [11], the authors demonstrated a residual capacity to activate large integrative networks in some of the MCS patients. Moreover, stimuli with emotional valence (cries and names) were able to induce in MCS patients a much more widespread activation than did meaningless noise [12], suggesting the relevance of speech content for higher-order auditory processing in MCS. Exceptionally, VS patients show higher atypical level of cortical brain activation, which was proposed as a surrogate marker of good prognosis [15].

Using resting state functional magnetic resonance imaging connectivity analyses, it has been shown that the default mode network connectivity was negatively correlated with the degree of clinical consciousness impairment, ranging from MCS patients over VS patients to coma patients [16, 17].

We present here the results of a study, in which fMRI was used in the evaluation of a relatively large number of patients with clinical diagnosis of *VS* or MCS. The aim was to determine, through fMRI-related brain activity, whether the status of wakefulness in VS is also accompanied by partial awareness, as occurs in MCS.

## MATERIALS AND METHODS

### Study Population

We studied 50 patients (age range: 27-58 years) from four to seven months after a brain injury (see Table **1**) for clinical and demographic details). By using the clinical international criteria for VS [18] and MCS [19] and validated behavioural scales such as the Glasgow Coma Scale (GCS) [20] and the Clinical Unawareness Assessment Scale (CUAS) [21], the patients were grouped into VS (n=23) and MCS (n=27) at the start of the study. GCS gives a reliable and objective way of recording the conscious state of a subject and its scoring system ranges between from 3 in case of deep unconsciousness to either 14 (original scale) or 15 (the more widely used modified or revised scale) in case of preserved consciousness. CUAS is a structured, systematic clinical approach for the assessment of awareness in which the three major sensory systems (auditory, visual, and somatic) and the motor system are assessed to establish whether some sensory stimuli can enter the central nervous system and the output of the motor pathway is functioning.

Six months after the fMRI examination, all patients were reassessed with Glasgow Outcome Scale (GOS) [22] and Revised Coma Recovery Scale (CRS-R) [23]. The GOS, which has a 5-point scoring system, represents a very global assessment of the general functioning of patients with traumatic brain injury (TBI) at some point of their recovery. The CRS-R was specifically developed to differentiate VS from MCS and to identify patients who have emerged from MCS. Its administration and scoring scheme explicitly incorporate the current diagnostic criteria for VS and MCS and it is unique in establishing a diagnosis and outcome directly from the examination findings. The basic structure of the CRS-R is similar to the GCS but its subscales (auditory, visual, motor, oromotor/verbal, communication, and arousal) are much more detailed, targeting more subtle signs of recovery of consciousness.

The present study was approved from the local Ethics Committee and written informed consent was obtained from the legal guardian of all patients.

### Conventional and Functional MRI Examination

All the subjects were examined using an identical MR protocol, which included combined conventional MRI/fMRI examination of the brain. No neuromuscular blockade or anesthetic drugs were used during scanning. Brain images were acquired using a 1.5 T scanner (Sonata Siemens, Erlangen, Germany). A sagittal survey image was used to identify the anterior commissure (AC) and posterior commissure (PC). A dual-echo, turbo spin-echo sequence (TR/TE1/TE2 = 2075/30/90 ms, with 256x256 matrix, 1 signal average, 250 mm field of view [FOV], 25 contiguous 5mm-thick slices), yielding PD and T2-weighted (T2-W) images was acquired in the axial plane, parallel to the AC-PC line. FLAIR images (TR= 9000 ms; TE = 150 ms; 50 contiguous 3-mm thick slices) were also acquired.

Blood oxygen level dependent (BOLD) contrast echo planar imaging (EPI) images were also acquired (TR = 3000 ms; TE = 40 ms, 25-mm FOV; 64x64 matrix for 10 contiguous 7mm-thick axial slices). A digitally recorded and adapted affective speech by a first-degree relative was administered to all study subjects through MRI-compatible noise attenuated headphones.

For this purpose, a domestic story segment of 150 sec duration was made out of “Little Red Riding Hood”. Two identical fMRI examinations were acquired by using a “block” design in which 30 seconds of auditory stimuli was alternated with a 30-second rest period for a total of 5 paired blocks. This acquisition protocol was similar to that reported by Monti *et al.* [24].

At the end of fMRI acquisition, whole brain structural images were acquired using a T1-W sequence (TR = 20 ms, TE = 3 ms, flip angle = 20º, 25 contiguous 5mm-thick axial slices parallel to the AC-PC line).

Data were analyzed using image analysis tools from the FMRIB Software Library (FSL v4.0) (http://www.fmrib.ox.ac.uk/fsl). Before statistical analysis, motion correction using MCFLIRT (Jenkinson M, 2001), non-brain removal using BET (Brain Extraction Tool) [25], spatial smoothing (Gaussian kernel of 5 mm full width at half maximum [FWHM]), intensity normalization, and non-linear high pass temporal filtering (Gaussian-weighted least-squares straight line fitting, with sigma= 75.0 s) were applied. Registration of fMRI data to structural T1-W images and standard space (MNI152 brain) was carried out using FLIRT (FMRIB’s Linear Image Registration Tool) [24, 26]. No structurally distorted brains were present that affected registration and spatial normalization to standard space.

### Statistical Analysis

Voxelwise statistical analyses were performed in the general linear model (GLM) framework. FMRIB’s Improved Linear Model (FILM) with local autocorrelation correction [27] was used to generate activation maps expressing relative signal change in active versus rest blocks. Group analysis was carried out using FLAME (FMRIB’s Local Analysis of Mixed Effects) stage 1 with automatic outlier detection [27]. One-sample T-test was used for mean activation within each group. At F-contrast testing among the groups of “VS stable”, VS converted to MCS (“VS converted”) and MCS, the main effect of group (*i.e.*, showing the brain regions with significant heterogeneity among the three patient groups) was obtained and used to mask all subsequent pairwise comparisons.

Clusters were detected on statistic images at a threshold of Z (Gaussianized T)>2.3. A corrected (for multiple comparisons across space) probability threshold of p=0.01 was applied to determine significant clusters.

Anatomical location of the local maxima within significant clusters was determined by reference to the Harvard-Oxford brain atlas, integrated into FSLView (also part of FSL).

## RESULTS

**Fig. (1):**
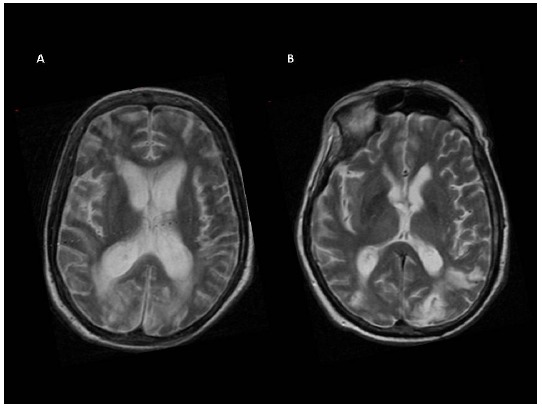
Illustrative example of T2-weighted axial.

Conventional MRI, performed at the time of fMRI, showed focal and diffuse brain signal abnormalities (Table **1** and Fig. **1**).

At clinical follow-up, performed six months after fMRI examination, 10 VS patients showed significant clinical improvement, as reported by significant differences in GOS and CRS scores with respect to the other VS patients (p<0.01) Table (**1**). In fact, they could repeatedly follow command to raise arms and head and could track visual stimuli with their eyes, indicating an vegetative (“VS stable”), and all the patients with MCS remained minimally conscious.

At baseline, significant brain fMRI activation during the acoustic stimuli was found bilaterally in primary auditory cortex (Heschl’s Gyrus [HG]: local maxima -43, -28, 8 mm and 47, -17, 7 mm for “VS stable” patients; -44, -30, 11 mm and 49, -20, 9 mm for “VS converted” patients; -42, -31, 11 mm and 48, -18, 9 mm for MCS patients) and associative auditory cortex (temporal planum [TP]: local maxima -50, -30, 10 mm and 51, -19, 9 mm for “VS stable” patients; -42, -27, 14 mm and 44, -26, 12 mm for “VS converted” patients; -43, -28, 12 mm and 45, -27, 13 mm for MCS patients).

Interestingly, the 10 “VS converted” patients, all suffering of TBI, showed a pattern of brain activation qualitatively very similar to that of the MCS patients, involving primary auditory cortices (HG) and extending to higher-order associative auditory cortex (TP) Fig. (**2**). Significant differences in brain activation were found among the three groups of “VS stable”, “VS converted” and MCS in the primary and supplementary auditory areas. Only the post-hoc contrasts where both “VS converted” and MCS patients were compared with “VS stable” patients and where “VS converted” patients were compared to MCS patients were significant Fig. (**3**). In particular, both “VS converted “and MCS patients had higher activation than “VS stable” patients in the primary auditory cortex (anterior transverse temporal gyrus, bank of the lateral sulcus on the dorsal surface of the temporal lobe), medially in the parainsular area and laterally in the posterior transverse temporal gyrus. “VS converted” patients showed lower activation than MCS patients only in the posterior transverse temporal gyrus. In particular, the correlation between fMRI activations (voxel cluster activation) and the clinical improvement for the 10 VS converted patients, showed a close relationship between primary and supplementary auditory areas and CRS-R (p<0.01).

## DISCUSSION

This is the first whole brain voxelwise study where fMRI was used to determine the incidence of undetected awareness in subjects with DOC, identifying three groups of patients: “VS stable”, “VS converted” and MCS.

After auditory stimuli, the three groups of patients showed brain activation not only in the primary auditory cortices but also in the associative cortices. However, this activation was higher in “*VS* converted” (to MCS) than in “VS stable” patients and in MCS when compared to “VS converted” patients. In particular, of the 50 patients examined, 10 patients originally classified as VS (“VS converted” patients) and all the 27 MCS patients showed, when compared to “VS stable” patients, higher activation mainly in the primary auditory cortex (anterior transverse temporal gyrus, bank of the lateral sulcus on the dorsal surface of the temporal lobe), medially in the parainsular area and laterally in the posterior transverse temporal gyrus. In addition, “VS converted” showed lower activation than MCS patients in the posterior transverse temporal gyrus.

Interestingly, six months after fMRI examination, “VS converted” patients were comparable to MCS on clinical ground. Indeed, in these patients, the assessment at bedside revealed some behavioral evidence of awareness, a finding that underscores the importance of thorough clinical examination for reducing the rate of misdiagnosis in such patients.

On the basis of these findings, some author could argue that these patients could be classified as affected by functional locked-in syndrome (FLI). The term ‘functional locked-in syndrome’ has been proposed to describe patients with a dissociation between extreme motor dysfunction and preserved higher cortical functions identified only by functional imaging techniques [28]. Nevertheless, patients clinically diagnosed in *VS* who are able to perform mental imagery tasks are still considered in the *VS* with preserved islands of consciousness, not as having functional locked-in syndrome. Formisano *et al.* [29], focused the attention on the topic that the patients with residual cognitive functions who are able to perform complex mental imagery tasks or show intentional communication ability should be diagnosed with functional locked-in syndrome and not *VS*. Our 10 patients are not be able to show intentional communication ability: in fact we described these subjects as “converted VS”.

The presence of anatomically appropriate brain activations in response to stimuli in VS patients has been linked with a positive outcome. A recent review of eight PET studies and six fMRI studies including a total of 48 VS patients estimated that the activation of high-level associative cortical regions can predict recovery of consciousness with a specificity of 93% and a sensitivity of 69% [15]. The presence of an fMRI activation pattern similar to MCS in the 10 VS patients reported in the current study is consistent with this trend. Functional neuroimaging studies indicate that some patients who appear unresponsive at the bedside may actually retain higher levels of self and environmental awareness, suggesting that fMRI activation patterns may provide relevant information on the residual cognitive function of such patients.

Using a silent picture-naming task to capture internal speech in DOC patients [30], strong language network activation was observed in a patient with locked-in syndrome, a patient who had emerged from MCS, and two MCS patients. One patient with *VS* also showed widespread activation of the language network, including the superior temporal, inferior frontal, and medial frontal gyri. In addition, patients with higher CRS-R scores showed more complete activation of language structures.

In another study on an MCS patient, passive listening and active auditory target detection tasks activated fronto-parietal networks similarly to healthy subjects [31].

In a recent study [24] on 54 patients, five TBI patients were able to modulate brain activity by generating voluntary, reliable, and repeatable BOLD responses in predefined neuroanatomical regions when prompted to perform imagery tasks. No such response was observed in any of the patients without TBI. The results of this study underline the potential for fMRI to bridge the dissociation occurring between behaviour that is readily observable during a standardized clinical assessment and the actual level of residual cognitive function after serious brain injury.

We included a relatively high number of DOC patients, which were studied for a long period of time. In fact, at present, there are three other longitudinal neuroimaging studies of recovery from DOC which have provided insights into the pathogenic mechanisms, reporting two VS patients [9, 32] and one MCS patient [33].

We enrolled here a similar number of DOC patients as a previous one [31], but, in addition, the present study differs because is the first that combined fMRI and clinical data to evaluate the cerebral and clinical changes of VS patients from the time of VS through the recovery.

In general, the findings of fMRI studies suggest a number of potential clinical and rehabilitative applications. Although clinical examination at bedside remains the mainstay for diagnosis, fMRI brain activation patterns may provide an adjunctive diagnostic role when behavioural findings are very limited or ambiguous. fMRI activation patterns may also inform prognosis in patients who show no behavioural evidence of language or visual processing. The future of diagnostic and prognostic assessment of DOC patients envisions a battery of neurobehavioral and neuroimaging techniques that serve as complementary clinical tools helping to differentiate the effects of underarousal, sensory impairment, motor dysfunction, and cognitive disturbance in the search for potential causes of behavioural unresponsiveness [34].

In addition to fMRI studies, neurophysiological approaches [5, 6, 35, 36], showed the role of low-resolution brain electromagnetic tomography, visual fixation, laser evoked potentials and neurosensorial stimulation, to assess a better differential diagnosis and prognostic marker in DOC field.

However, fMRI could be a really potentially reliable marker for differential diagnosis and prognosis. Active paradigm seems to provide a valuable additional diagnostic tool in cases of patients with atypical presentation. Negative results, however, must be cautiously interpreted in case of patients with severely altered level of vigilance, who could present only transient activity in response to the stimulus presentation.

Our study emphasizes that functional neuroimaging might subcategorize the clinical entities of VS and MCS, thus providing an important aid to the differentiation of DOC conditions.

Although further research is necessary to better understand the clinical meaning of the cortical activations in higher-order networks observed in ten of our DOC patients (“VS converted”), our study included a reasonable high number of patients and is the first study which performed a clinical follow-up to address the prognostic value of an fMRI paradigm.

### Compliance with Ethical Standards

The authors declared no potential conflicts of interest.

The present study was approved from the local Ethics Committee and written informed consent was obtained from the legal guardian of all patients. All procedures performed in this study were in accordance with the ethical standards of the institutional and national research committee and with the 1964 Helsinki declaration and its later amendments or comparable ethical standards.

This study was funded by Italian Health Minister (grant number GR-2013-02359341).

## Figures and Tables

**Fig. (2):**
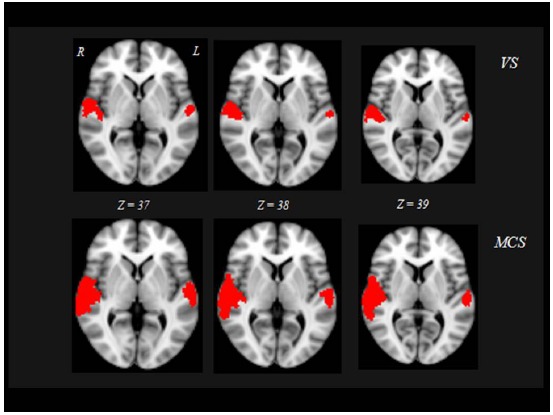
Red shows activated brain areas (p<0.01, cluster corrected for multiple comparisons) during acoustic stimuli in “VS stable” (A), and MCS (B) patients, overlaid on the MNI standard brain. Z coordinates are expressed in mm.

**Fig. (3):**
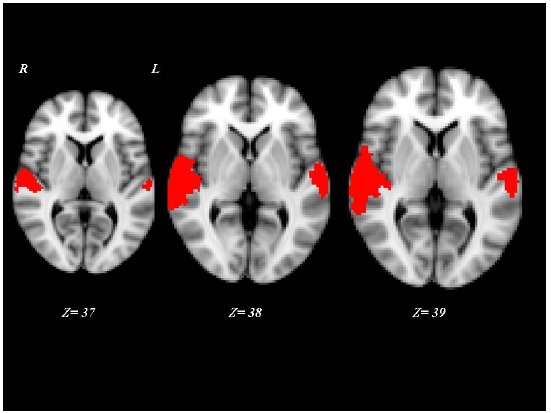
Post-hoc contrasts where both “VS converted” were compared with MCS and “VS converted”. Red shows activated brain areas (p<0.01, cluster corrected) as results of a subtraction analysis among activation VS stable, MCS and VS converted patients. Z coordinates are expressed in mm.

**Table 1:** Clinical-demographic and conventional MRI findings in VS and MCS patients.

**Patients**	**Age** **(years)**	**GCS (baseline)**	**CUAS (baseline)**	**GOS** **(at 6 months)**	**CRS-R** **(at 6 months)**	**Cause of DOC** **(n. of patients)**	**MRI findings (n. of patients)**
VS stable (n. 13)	51±2.1	8±1	4±1	3±1	6±1	TBI: n. 6 ABI: n. 3 CVA: n. 4	Diffuse signal abnormalities: n. 6 Right frontal focal abnormalities: n. 3 Left frontal focal abnormalities: n. 1 Left fronto-parietal focal abnormalities: n. 3
VS converted (n. 10)	48±1.2	8±1	4±1	2±1	9±1	TBI: n. 10	Diffuse signal abnormalities: n. 6 Right frontal focal abnormalities: n. 2 Left frontal focal abnormalities: n. 2
MCS (n. 27)	52±6.1	11±1	6±1	2±1	10±1	TBI: n. 15 ABI: n. 5 CVA: n. 7	Diffuse signal abnormalities: n. 16 Left frontal focal abnormalities: n. 7 Right fronto-parietal focal abnormalities: n. 4

**Abbreviations**: VS: Vegetative State; MCS: Minimally Conscious State; GCS: Glasgow Coma Scale; CUAS: Clinical Unawareness Assessment Scale; GOS: Glasgow Outcome Scale; CRS-R: Coma Recovery Scale-Revised; DOC: Disorders of Consciousness; TBI: Traumatic Brain Injury; ABI: Anoxic Brain Injury; CVA: CerebroVascular Accident.
